# Adapted Murine Sepsis Score: Improving the Research in Experimental Sepsis Mouse Model

**DOI:** 10.1155/2022/5700853

**Published:** 2022-01-27

**Authors:** Maicon Machado Sulzbacher, Lucas Machado Sulzbacher, Felipe Rafael Passos, Bruna Letícia Endl Bilibio, Kauana de Oliveira, Wellington Felipe Althaus, Matias Nunes Frizzo, Mirna Stela Ludwig, Ivana Beatrice Mânica Da Cruz, Thiago Gomes Heck

**Affiliations:** ^1^Research Group in Physiology, Regional University of Northwestern Rio Grande do Sul State (UNIJUÍ), Ijuí, RS, Brazil; ^2^Postgraduate Program in Integral Attention to Health (PPGAIS-UNIJUÍ/UNICRUZ), Ijuí, RS, Brazil; ^3^Postgraduate Program in Pharmacology, Federal University of Santa Maria (UFSM), Brazil; ^4^Postgraduate Program in Mathematical and Computational Modelling (UNIJUI), Brazil

## Abstract

The Murine Sepsis Score (MSS) is used to assess the severity of sepsis in rats and mice based on observational characteristics. The quantitative variables of glycemia, body weight, and temperature are predictors of severity in experimental models of sepsis. Therefore, our study sought to adapt the MSS with the same variables to indicate earlier the severity of the disease in murine models of the disease. Sepsis mice presented hypoglycemia, weight loss, and hypothermia. Therefore, these variables were included in the Adapted Murine Sepsis Score (A-MSS). The A-MASS presented 100% specificity and 87.5% sensibility been able to differentiate the early sepsis symptoms and its severity. The A-MSS allows an early and more complete diagnosis of sepsis in mice and might be considered as a procedure to improve the analysis of systemic sepsis dysfunction in murine experimental models.

## 1. Introduction

Sepsis is a systemic infection characterized by multiple organ dysfunction and high mortality rates. Therefore, studies investigating the pathophysiology in sepsis are required [1, 2], and the use of experimental mouse models has been considered a fundamental approach [3], due to the physiological similarities between human and mice, including a great genotypic homology in terms of hormonal, cell signaling, receptors, immune system, and oxidative and cell stress responses [4]. The bacteria-induced sepsis in animal models has similar characteristics found in septic patients [5, 6] due to the gram-negative bacteria membrane endotoxins like lipopolysaccharide (LPS) and lipoteichoic acid (LTA) from gram-positive bacteria [7].

The pathogen-associated molecular patterns (PAMPS) are recognized by cell surface receptors, for example, Toll-like receptors, expressed by the human and animal leukocytes. In this way, LPS is recognized by the host cell defense by TRL4, whereas TLR2 receptors recognize LTA. Both molecular interactions induce the activation of the nuclear kappa B transcription factor (NF-*κ*B), which initiates the synthesis of several proinflammatory cytokines. Also, these PAMPS may interact with platelets and endothelial tissue, adding microvascular complications that have an essential role in the inflammation and systemic dysfunction in sepsis [8].

One of the leading infectious focus in sepsis is abdominal origin. *Staphylococcus aureus* and *Escherichia coli* bacteria are commensals of the mouse gastrointestinal tract and can be found in the feces of these *animals* [5, 9]. One of the leading infectious focus in sepsis is abdominal origin. *Staphylococcus aureus* and *Escherichia coli* bacteria are commensals of the mouse gastrointestinal tract and can be found in the feces of these animals [5, 9]. Therefore, an experimental model of sepsis is peritonitis induction by the intraperitoneal administration of the autogenous fecal solution. From this procedure, sepsis is recognized by an application of Murine Sepsis Score (MSS) that consists of observing and categorizing the level of consciousness, activity, behavior, response to stimuli, respiratory rate, and quality of breathing movements [10]. However, it is possible to observe a decreased glycemic levels, core temperature, and body weight loss if we follow up animals for 24 hours [11, 12], but these variables have not been included in the MSS protocol [10].

Patients and animals with sepsis may present hypothermia [13, 14], hypoglycemia [11, 15], body weight loss [16], and hematological alterations [17]. Also, early identification of sepsis is a well-known, relevant procedure to allow successful treatment of the disease [18]. Therefore, herein, we proposed an adaptation in the MSS (A-MSS) to provide a score that identifies sepsis in an experimental model in the initial stages.

## 2. Material and Methods

### 2.1. Animals

Fifteen male C57BL/6 mice aged 90 to 150 days from the Life Sciences Department (DCVida) of the Northwestern Regional University of Rio Grande do Sul State (UNIJUÍ) were used in this study.

### 2.2. Ethics Statement

The ethical principles established by the International Animal Protection Standards have been respected [19], Brazilian Code of Animal Experimentation—1988, as well as the National Institutes of Health (NIH) Guide to Laboratory Animal Care and Use. This study was approved by the Animal Use Ethics Committee of UNIJUÍ (CEUA 048/2016).

### 2.3. Experimental Design

The animals were divided into two experimental groups: control (*n* = 7) and sepsis (*n* = 8). A 20% fecal solution (200 mg/mL) was prepared with fresh stool in 0.9% NaCl, and it was administered at a dose of 5 *μ*L/g (1 mg/g, i.p.) in the sepsis group, while control animals received 0.9% NaCl [12]. The animals were monitored for glycemia, blood count, rectal temperature, and MSS (Table S1), before (time zero) and at 4, 12, and 24 hours after sepsis induction.

### 2.4. Procedures Details

Glycemia was measured by distal tail puncture (~5 *μ*L) using Optium Xceed® glucometer. Rectal temperature was measured with a digital thermometer, and body weight was measured using a semianalytical balance. For hematological analysis, blood was collected (0, 4, 12, and 24 h) by caudal puncture (10 *μ*L). Samples were diluted 1 : 3 with 0.9% saline and 1 *μ*L of anticoagulant (EDTA). Hematological strains were performed on the slide for differential leukocyte count, stained with a panoptic type [20].

### 2.5. Statistical Analysis

Data were analyzed using the Kolmogorov-Smirnov test. The results in which the evaluation was performed over time were verified by two-way ANOVA (time × treatment) followed by Bonferroni posttest. Spearman or Pearson correlation test was performed. The potential diagnosis of A-MSS within four hours after sepsis induction was tested by ROC curve. The cutoff point for A-MSS diagnosis was determined by observing the most appropriate balance between sensitivity and specificity in the ROC curve [21]. The significance level of 5% (*P* < 0.05) was considered.

## 3. Results

**Figure 1:**
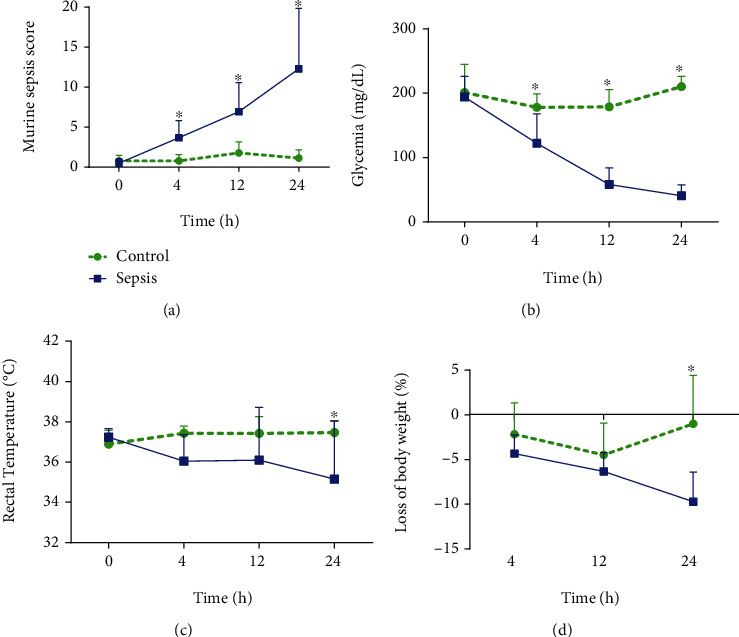
Measurement of Murine Sepsis Score (MSS) (^∗^*P* < 0.0001; *F*_1.52_ = 31.43) (a), glycemia (^∗^*P* < 0.0001; *F*_1.52_ = 124.3) (b), rectal temperature (^∗^*P* < 0.0001; *F*_1.130_ = 8.23) (c), and body weight loss (^∗^*P* < 0.0002; *F*_1.39_ = 16.95) (d) within 24 hours after induction of sepsis with 20% fecal solution (1 mg/g). Statistical analysis was performed by two-way ANOVA followed by *Bonferroni* posttest.

The animals submitted to sepsis presented increased MSS 12 h and 24 h after fecal solution administration (Figure 1(a)). Also, animals with sepsis showed a decrease in glycemia, rectal temperature, and body weight (Figures 1(b)–1(d)). It was possible to verify that sepsis induces alterations in appearance, level of consciousness, activity, stimulus-response, eye aspect, and respiratory rate and quality (Supplementary Figure S1).

**Figure 2:**
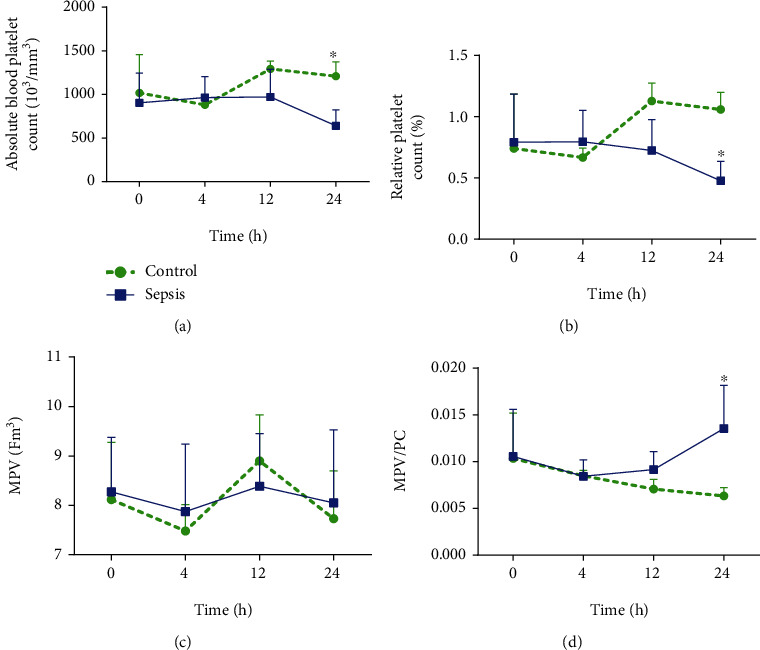
Absolute blood platelet count (^∗^*P* = 0.0021; *F*_1.48_ = 10.53) (a), relative platelet count (^∗^*P* = 0.013; *F*_1.40_ = 6.69) (b), mean platelet volume (MPV) (*P* = 0.6) (c), and MPV to platelet ratio (MPV/PC) (^∗^*P* < 0.0021; *F*_1.48_ = 10.53) (d), within 24 hours after induction of sepsis with 20% fecal solution (1 mg/g). Statistical analysis was performed using two-way ANOVA followed by a *Bonferroni* posttest.

Sepsis was able to cause a decrease in absolute and relative platelet count (Figures 2(a) and 2(b)) without inducing mean platelet volume alterations (MPV) (Figure 2(c)) but increased the MPV to platelet ratio (MPV/PC) (Figure 2(d)).

**Figure 3:**
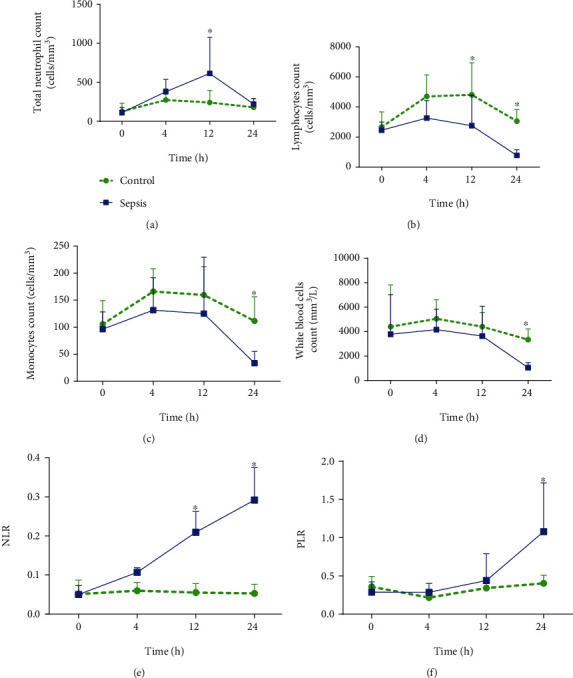
Total neutrophil count (^∗^*P* = 0.047; *F*_1.40_ = 4.17) (a), lymphocyte count (^∗^*P* = 0.0002; *F*_1.44_ = 16.62) (b), monocyte count (^∗^*P* = 0.024; *F*_1.40_ = 5.47) (c), white blood cell count (^∗^*P* = 0.0021; *F*_1.48_ = 3.34) (d), ratio between neutrophil-lymphocyte count (NLR) (^∗^*P* < 0.0001; *F*_1.4_ = 76.05) (e), and platelet-lymphocyte ratio (PLR) (^∗^*P* = 0.031; *F*_1.40_ = 4.95) (f), within 24 hours after induction of sepsis by 20% fecal solution (1 mg/g). Statistical analysis was performed using two-way ANOVA followed by *Bonferroni* posttest.

The neutrophil count increased in 12 h after sepsis induction, then returning to basal level in the next 12 hours (Figure 3(a)). At 24 h, we found a decrease in white blood cell count, including a decrease in lymphocyte and monocyte count (Figures 3(d), 3(b), and 3(c), respectively).

These alterations in immune cells, as well as observed in the platelets, impact in the neutrophil-lymphocyte ratio (NLR), which increased at 12 h together with the platelet to lymphocyte ratio (PLR) in 24 h (Figures 3(e) and 3(f), respectively).

**Figure 4:**
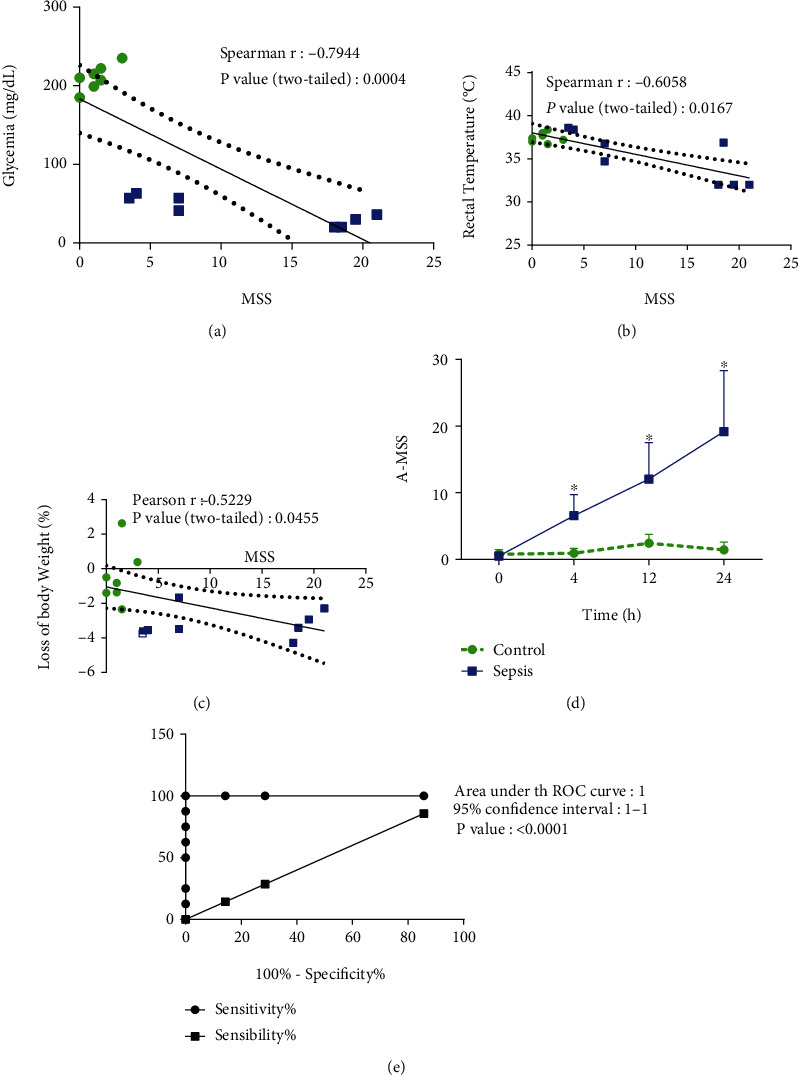
Correlation between MSS and glycemia (a), temperature (b) and relative weight loss (c), and 24 hours after sepsis induction and measurement of Adapted Murine Sepsis Score (A-MSS) throughout the experiment period (^∗^*P* < 0.0001; *F*_1.52_ = 58.39) (d), and ROC curve (e) at time 4 hours after induction of sepsis with 20% fecal solution (1 mg/g). Statistical analysis was performed using two-way ANOVA followed by *Bonferroni* posttest (d) and curve ROC (e).

A strong negative correlation (*r* = −0.79) was observed between MSS and glycemia 24 hours after sepsis induction. Also, a moderate negative correlation was observed between MSS and body temperature (*r* = −0.61) and weight loss (*r* = −0.52) in the same time-point (Figures 4(a)–4(c)). Therefore, we proposed the A-MSS which was able to report sepsis within four hours of the sepsis induction (Figure 4(d)). When this score was evaluated from the ROC curve in this period, it was found that it can be used as a diagnostic standard (Figure 4(e)), from an A-MSS value of 3.5, obtaining a sensitivity of 87.5% and specificity of 100%.

The proposed A-MSS is detailed in Table 1. A-MSS measured four hours after sepsis induction showed strong negative correlation with the severity marker parameters in experimental sepsis: lymphocyte count (*r* = −0.79), white blood cells count (*r* = −0.83), and platelet count (*r* = −0.77) (Supplementary Figure S2A-C). Also, there was a strong positive correlation between A-MSS and NLR (*r* = 0.74) (Supplementary Figure S2D). There is no statistically significant correlation between A-MSS and platelet to lymphocyte ratio (*P* > 0.05) and also between A-MSS and MPV/PC ratio (*P* > 0.05).

## 4. Discussion

We showed that the inclusion of variables easily measurable as glycemia, temperature, and body weight in the MSS may improve the research in sepsis mice model. Our proposal of A-MSS represents a sum of observations, and together with the established MSS, the abovementioned variables might be considered as a new score for the evaluation of sepsis in experimental models (Table 1).

Sepsis is a complex disease that requires a complex form to diagnose it successfully [22]. The A-MSS added parameters to the MSS, which allowed an indirect assessment of the cardiovascular (temperature) and metabolic systems (glycemia and body weight), which are essential for the prognosis in sepsis in animals and humans. In humans, the diagnosis is made by measures of neurological, cardiovascular, respiratory, renal, hepatic, and platelet dysfunction, by the reproducible Sequential Organ Failure Assessment (SOFA) score [23]. We believe that A-MSS approximates the clinical variables evaluated in SOFA for the early diagnosis of sepsis in animal models.

The central nervous system dysfunction is mainly characterized by septic encephalopathy, followed by autonomic failure [24]. These dysfunctions cause tissue damage and impair brain function [25]. In our study, these neurological effects were reflected in the impairment of the level of consciousness and activity in the sepsis group (Figure S1A, C). The neurological symptoms can be identified in the first hours by MSS [13], but using the A-MSS allows the assessment of autonomic insufficiency evaluating the temperature (Figure 1(c)).

Neurohypophysial dysfunction promotes decreased hepatic gluconeogenesis-promoting adrenocortical hormones and liver and muscle glycogenolysis, causing a decrease in glycemia [26]. Furthermore, LPS is able to decrease the activity of hepatic and renal enzyme phosphoenolpyruvate carboxykinase (PEPCK) promoting gluconeogenesis and hypoglycemia [27], as observed in Figure 1(b).

Sepsis impairs neuroendocrine regulation by neuronal dysfunction impairing the secretion of vasopressor hormones (NASCIMENTO et al., 2017), accompanied by the desensitization of receptors for vasoconstriction [28]. Also, LPS and cytokines induce the nitric oxide production by macrophages, neutrophils, and monocytes [28]. Decreased cardiac output and blood pressure may be associated with hypothermia (Figure 1(c)) [13, 29], reinforcing the need to measure body temperature in sepsis mouse models.

A catabolic condition leads to a reduction in muscle mass [30] is related to increased hospitalization period and mortality [16]. This metabolic effect is also verified in an experimental sepsis model [11, 12, 31], and the results found in our study (Figure 1(d)) may be associated with muscle atrophy and lipid catabolism [32]. Also, leukocytes release *interleukin*-*1 beta*, which has a direct effect on appetite inhibition and food intake, reflecting in body weight loss [33].

The platelet function disorders play a crucial role in the pathophysiology of sepsis, with prognostic accuracy [34]. LPS and cytokine endotoxemia (tumor necrosis factor-alpha (TNF-*α*), IL-8, IL-15) stimulate endothelium, monocytes, neutrophils, and basophils to secrete platelet-activating factor (PAF) [34]. PAF triggers platelet aggregation with the formation of microthrombi, which, together with leukocyte-induced hemophagocytosis [35], may result in decreased total platelet count (thrombocytopenia) and its relative rate [36]. Regarding MPV, it is already known that the 24 h period is insufficient for the increase in MPV to indicate the severity in patients with sepsis [37], as observed in our study (Figure 2(c)). It has been proposed that MPV indicates severity in just after 72 hours [38] and that the evaluation of MPV is insufficient to predict the worsening of sepsis due to peritonitis with gram-negative bacteria [17]. The dysfunction in the platelet count can also be observed by analyzing the ratios between the MPV and platelet count (MPV/PC), representing the risk of microthrombi formation [39]. The MPV/PC is a predictor of worsening of sepsis, specifically when it accuses the systemic infection with gram-negative bacteria [17], as we observed in our study (Figure 2(d)).

Immunosuppression observed by decreasing lymphocyte and monocytes leads to a decrease in total leukocytes (Figures 3(b)–3(d)). On the other hand, the bone marrow has a reservoir of neutrophils that are released into the circulation to combat infectious in the peritoneal cavity, as verified 12 h after sepsis induction [40]. Neutrophils are immune cells of the first line of defense against bacterial infection and may suffer exacerbated apoptosis in severe sepsis [17].

Studies suggest the application of NLR and PLR as inflammatory biomarkers [17]. PLR and NLR elevation is a prognostic marker of lethality in patients with peritonitis [17], as well as in an animal models [41], similarly to Figures 3(e) and 3(f). Also, platelet and immune biomarkers may indicate etiological agents [17]. Thus, the A-MSS proposed in our study was able to indicate sepsis in mice just after four hours, correlated with the 24-hour values of the biomarkers mentioned above: lymphocyte count, white blood cell count, platelet count, and NLR. These pieces of evidence allow proposing the A-MSS as a relevant tool for murine severe infectious diseases since it was able to predict the severity systemic dysfunction of the mouse sepsis model.

## 5. Conclusion

The A-MSS allows an early and more complete diagnosis of sepsis in mice and might be considered as a procedure to improve the analysis of systemic sepsis dysfunction in mice. The inclusion of new variables that can be directly measured represents the inclusion of objective criteria in a quite subjective exam to improve the accuracy of studies in mouse severe sepsis model.

## Figures and Tables

**Table 1:** Adapted Murine Sepsis Score (A-MSS).

Score	0	1	2	3	4
Appearance	Coat is smooth	Patches of hair piloerected	Majority of back is piloerected	Piloerection may or may not be present; mouse appears “puffy”	Piloerection may or may not be present; mouse appears emaciated
Level of consciousness	Mouse is active	Mouse is active but avoids standing upright	Mouse activity is noticeably slowed. The mouse is still ambulant	Activity is impaired. Mouse only moves when provoked; movements have a tremor	Activity severely impaired. Remains stationary when provoked, with possible tremor
Activity	Normal amount of activity. Mouse is any of eating, drinking, climbing, running, and fighting	Slightly suppressed activity. Mouse is moving around bottom of cage	Suppressed activity. Mouse is stationary with occasional investigative movements	No activity	No activity. Mouse experiencing tremors, particularly in the hind legs
Response to stimulus	Mouse responds immediately to auditory stimulus or touch	Slow or no response to auditory stimulus; strong response to touch (moves to escape)	No response to auditory stimulus; moderate response to touch (moves a few steps)	No response to auditory stimulus; mild response to touch (no locomotion)	No response to auditory stimulus. Little or no response to touch. Cannot right itself if pushed over
Eyes	Open	Eyes not fully open, possibly with secretions	Eyes at least half closed, possibly with secretions	Eyes half closed or more, possibly with secretions	Eyes closed or milky
Respiration rate	Normal, rapid mouse respiration	Slightly decreased respiration (rate not quantifiable by the eye)	Moderately reduced respiration (rate at the upper range of quantifying by the eye)	Severely reduced respiration (rate easily countable by the eye, 0.5 s between breaths)	Extremely reduced respiration (>1 s between breaths)
Respiration quality	Normal	Brief periods of laboured breathing	Laboured, no gasping	Laboured with intermittent gasps	Gasping
Rectal temperature (°C)	36-38	> 38	< 36- ≥ 35	< 35- ≥ 34	< 34
Glycemia (mg/dL)	≥148	≤148-≥122	<122-≥58	<58->40	≤ 40
Relative body weight loss (%)	0-5	5-10	10-15	15-20	> 20

## Data Availability

All data will be available under request for authors.

## Abbreviations

LPS: Lipopolysaccharide
LTA: Lipoteichoic acid
MSS: Murine Sepsis Score
A-MSS: Adapted Murine Sepsis Score
NaCl: Sodium chloride
ROC: Receiver operating characteristic
MPV: Mean platelet volume
MPV/PC: Ratio between MPV and platelet count
NLR: Neutrophil-lymphocyte ratio
PLR: Platelet to lymphocyte ratio
M-CASS: Mouse clinical assessment score for sepsis
SOFA: Sequential Organ Failure Assessment
CNS: Central nervous system
PEPCK: Phosphoenolpyruvate carboxykinase
iNOS: Inducible nitric oxide synthase
NO: Nitric oxide
IL-1*β*: *Interleukin*-*1 beta*
TNF-*α*: Tumor necrosis factor alpha
PAF: Platelet-activating factor.

## Additional Points

*Limitations*. Our study did not use laboratory tests for sepsis evaluations such as bilirubin, creatinine, or lactate.

## Supplementary Materials

Supplementary 1 Table S1: Murine Sepsis Score (MSS).

Supplementary 2 Figure S1: individualized MSS components, appearance, level of consciousness, activity, response to stimulus, aspect of eyes, respiratory frequency, and respiratory quality (A-G) within 24 hours after induction of sepsis with 20% fecal solution (1 mg/g).

Supplementary 3 Figure S2: correlation between A-MSS four hours after induction and lymphocyte count, total leukocytes, platelets, and the ratios between neutrophils and lymphocytes (NLR), platelets and lymphocytes (PLR), and mean platelet volume and platelet count (MPV/PC) (A-F) within 24 hours after induction of sepsis with 20% fecal solution (1 mg/g).

## References

[1] Meurer M., Höcherl K. (2019). Endotoxaemia differentially regulates the expression of renal Ca2+ transport proteins in mice. *Acta Physiologica*.

[2] Skirecki T., Cavaillon J.-M. (2019). Inner sensors of endotoxin – implications for sepsis research and therapy. *FEMS Microbiology Reviews*.

[3] Remick D. G., Ayala A., Chaudry I. (2019). Premise for standardized sepsis models. *Shock*.

[4] Dutta S., Sengupta P. (2016). Men and mice: Relating their ages. *Life Sciences*.

[5] Holtfreter S., Radcliff F. J., Grumann D. (2013). Characterization of a mouse-adapted Staphylococcus aureus strain. *PloS one*.

[6] Parker S. J., Watkins P. E. (2001). Experimental models of gram-negative sepsis. *The British Journal of Surgery*.

[7] INSTITUTO LATINO AMERICANO DE SEPSE (2019). *Relatório Nacional: Protocolos Gerenciados de Sepse. São Paulo: [s.n.]. Disponível em*.

[8] Grin P. M., Dwivedi D. J., Chathely K. M. (2018). Low-density lipoprotein (LDL)-dependent uptake of gram-positive lipoteichoic acid and gram-negative lipopolysaccharide occurs through LDL receptor. *Scientific Reports*.

[9] Stromberg Z. R., Van Goor A., Redweik G. A., Wymore Brand M. J., Wannemuehler M. J., Mellata M. (2018). Pathogenic and non-pathogenic Escherichia coli colonization and host inflammatory response in a defined microbiota mouse model. *Disease models & mechanisms*.

[10] Shrum B., Anantha R. V., Xu S. X. (2014). A robust scoring system to evaluate sepsis severity in an animal model. *BMC Research Notes*.

[11] Sulzbacher M. M., Santos A. B., Basso R. D., Hirsh G. E., Ludwig M. S., Heck T. G. (2018). Efeitos do tratamento com glutamina via enteral em modelo animal de sepse. *Saúde (Santa Maria)*.

[12] Sulzbacher M. M., Sulzbacher L. M., Passos F. R. (2020). A single dose of eHSP72 attenuates sepsis severity in mice. *Scientific Reports*.

[13] Mai S. H., Sharma N., Kwong A. C. (2018). Body temperature and mouse scoring systems as surrogate markers of death in cecal ligation and puncture sepsis. *Intensive Care Medicine Experimental*.

[14] Tiruvoipati R., Ong K., Gangopadhyay H., Arora S., Carney I., Botha J. (2010). Hypothermia predicts mortality in critically ill elderly patients with sepsis. *BMC Geriatrics*.

[15] Park S., Kim D. G., Suh G. (2012). Mild hypoglycemia is independently associated with increased risk of mortality in patients with sepsis: a 3-year retrospective observational study. *Critical Care*.

[16] Auiwattanakul S., Chittawatanarat K., Chaiwat O. (2019). Effects of nutrition factors on mortality and sepsis occurrence in a multicenter university-based surgical intensive care unit in Thailand (THAI- SICU study). *Nutrition*.

[17] Djordjevic D., Rondovic G., Surbatovic M. (2018). Neutrophil-to-Lymphocyte Ratio, Monocyte-to-Lymphocyte Ratio, Platelet-to- Lymphocyte Ratio, and Mean Platelet Volume-to-Platelet Count Ratio as Biomarkers in Critically Ill and Injured Patients: Which Ratio to Choose to Predict Outcome and Nature of Bacteremia?. *Mediators of Inflammation*.

[18] Kumar A., Roberts D., Wood K. E. (2006). Duration of hypotension before initiation of effective antimicrobial therapy is the critical determinant of survival in human septic shock. *Critical Care Medicine*.

[19] Hoff C. (1980). Immoral and moral uses of animals. *New England Journal of Medicine*.

[20] Bedell S. E., Bush B. T. (1985). Erythrocyte sedimentation rate. From folklore to facts. *The American journal of medicine*.

[21] Almeida R. T., Almeida M. M., Araújo T. M. (2009). Obesidade abdominal e risco cardiovascular: desempenho de indicadores antropométricos em mulheres. *Arquivos Brasileiros de Cardiologia*.

[22] Raith E. P., Udy A. A., Bailey M. (2017). Prognostic accuracy of the SOFA score, SIRS criteria, and qSOFA score for in-hospital mortality among adults with suspected infection admitted to the intensive care unit. *JAMA*.

[23] Singer M., Deutschman C. S., Seymour C. W. (2016). The third international consensus definitions for sepsis and septic shock (Sepsis-3). *Journal of the American Medical Association*.

[24] VIANA, R. A. P. P (2013). *Sepse para enfermeiros*.

[25] Gasparotto J., Girardi C. S., Somensi N. (2018). RAGE mediates neurodegeneration in sepsis. *Journal of Biological Chemistry*.

[26] Pizarro C. F., Troster E. J. (2007). Adrenal function in sepsis and septic shock Função adrenal na sepse e choque séptico. *Jornal de Pediatria*.

[27] Caton P. W., Nayuni N. K., Murch O., Corder R. (2009). Endotoxin induced hyperlactatemia and hypoglycemia is linked to decreased mitochondrial phosphoenolpyruvate carboxykinase. *Life Sciences*.

[28] VELASCO, F. P. DA S. E I. T (2007). *Sepse*.

[29] Pinto C. F., Watanabe M., Fonseca C. D. ., Ogata C. I., Vattimo M. . F. F. (2012). A sepse como causa de lesão renal aguda: modelo experimental. *Revista da Escola de Enfermagem da U S P*.

[30] Langhans C., Weber-Carstens S., Schmidt F. (2014). Inflammation-induced acute phase response in skeletal muscle and critical illness myopathy. *PLoS One*.

[31] Xianchu L., Lan P. Z., Qiufang L. (2016). Naringin protects against lipopolysaccharide-induced cardiac injury in mice. *Environmental Toxicology and Pharmacology*.

[32] Al-Nassan S., Fujino H. (2018). Exercise preconditioning attenuates atrophic mediators and preserves muscle mass in acute sepsis. *General physiology and biophysics*.

[33] DO M. C., Kluger M. J., Vander A. J. (1985). Suppression of food intake during infection: is interleukin-1 involved?. *American Journal of Clinical Nutrition*.

[34] Bedet A., Razazi K., Boissier F. (2017). Mechanisms of thrombocytopenia during septic shock: a multiplex cluster analysis of endogenous sepsis mediators. *Shock*.

[35] Francois B., Trimoreau F., Vignon P., Fixe P., Praloran V., Gastinne H. (1997). Thrombocytopenia in the sepsis syndrome: role of hemophagocytosis and macrophage colony-stimulating factor. *The American journal of medicine*.

[36] Thiolliere F., Serre-Sapin A. F., Reignier J. (2013). Epidemiology and outcome of thrombocytopenic patients in the intensive care unit: results of a prospective multicenter study. *Intensive Care Medicine*.

[37] Sadaka F., Donnelly P. L., Griffin M. T., O’Brien J., Lakshmanan R. (2014). Mean platelet volume is not a useful predictor of mortality in septic shock. *Journal of Blood Disorders & Transfusion*.

[38] Kim C. H., Kim S. J., Lee M. J. (2015). An increase in mean platelet volume from baseline is associated with mortality in patients with severe sepsis or septic shock. *PLoS One*.

[39] Oh G. H., Chung S. P., Park Y. S. (2017). Mean platelet volume to platelet count ratio as a promising predictor of early mortality in severe sepsis. *Shock*.

[40] Hokama N. K., Machado P. E. (1997). Interpretação clínica do hemograma nas infecções. *Jornal Brasileiro de Medicina*.

[41] Brooks H. F., Osabutey C. K., Moss R. F., Andrews P. L., Davies D. C. (2007). Caecal ligation and puncture in the rat mimics the pathophysiological changes in human sepsis and causes multi-organ dysfunction. *Metabolic Brain Disease*.

[42] Santos-Junior N. N., Costa L. H., Catalão C. H., Kanashiro A., Sharshar T., Rocha M. J. (2017). Impairment of osmotic challenge-induced neurohypophyseal hormones secretion in sepsis survivor rats. *Pituitary*.

