# Supplementary material for: Adapted Murine Sepsis Score: Improving the Research in Experimental Sepsis Mouse Model
Source: Biomed Res Int. 2022 Jan 27;2022:5700853. doi: 10.1155/2022/5700853 (PMC8814713; doi:10.1155/2022/5700853)
Supplement: Supplementary 1 — Table S1: Murine Sepsis Score (MSS). [file 5700853.f1.docx]

| **Table S1. Murine Sepsis Score (MSS) (9)** | | | | | |
| --- | --- | --- | --- | --- | --- |
| **Score** | **0** | **1** | **2** | **3** | **4** |
| Appearance | Coat is smooth | Patches of hair piloerected | Majority of back is piloerected | Piloerection may or may not be present, mouse appears “puffy” | Piloerection may or may not be present, mouse appears emaciated |
| Level of consciousness | Mouse is active | Mouse is active but avoids standing upright | Mouse activity is noticeably slowed. The mouse is still ambulant | Activity is impaired. Mouse only moves when provoked, movements have a tremor | Activity severely impaired.Remains stationary when provoked, with possible tremor |
| Activity | Normal amount of activity. Mouse is any of: eating, drinking, climbing, running, fighting | Slightly suppressed activity. Mouse is moving around bottom of cage | Suppressed activity. Mouse is stationary with occasional investigative movements | No activity | No activity. Mouse experiencing tremors, particularly in the hind legs |
| Response to stimulus | Mouse responds immediately to auditory stimulus or touch | Slow or no response to auditory stimulus; strong response to touch (moves to escape) | No response to auditory stimulus; moderate response to touch (moves a few steps) | No response to auditory stimulus; mild response to touch (no locomotion) | No response to auditory stimulus. Little or no response to touch. Cannot right itself if pushed over |
| Eyes | Open | Eyes not fully open, possibly with secretions | Eyes at least half closed, possibly with secretions | Eyes half closed or more, possibly with secretions | Eyes closed or milky |
| Respiration rate | Normal, rapid mouse respiration | Slightly decreased respiration (rate not quantifiable by eye) | Moderately reduced respiration (rate at the upper range of quantifying by eye) | Severely reduced respiration (rate easily countable by eye, 0.5 s between breaths) | Extremely reduced respiration (>1 s between breaths) |
| Respiration quality | Normal | Brief periods of laboured breathing | Laboured, no gasping | Laboured with intermittent gasps | Gasping |

**Reference:**

9. Shrum B, Anantha R V., Xu SX, Donnelly M, Haeryfar SMM, McCormick JK, et al. A robust scoring system to evaluate sepsis severity in an animal model. BMC Res Notes [Internet]. 2014;7(1):1–11. Available from: BMC Research Notes
