# Supplementary figures and images for: Adapted Murine Sepsis Score: Improving the Research in Experimental Sepsis Mouse Model
Source: Biomed Res Int. 2022 Jan 27;2022:5700853. doi: 10.1155/2022/5700853 (PMC8814713; doi:10.1155/2022/5700853)

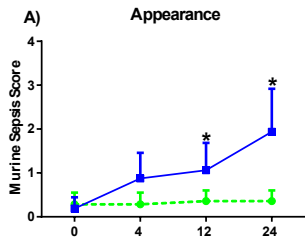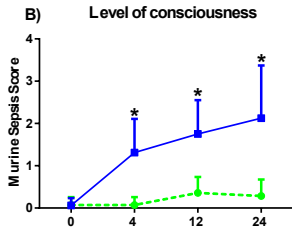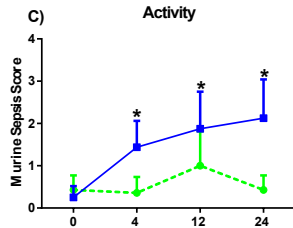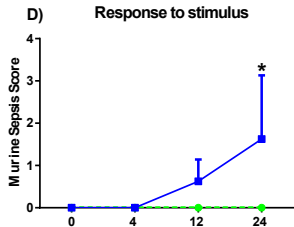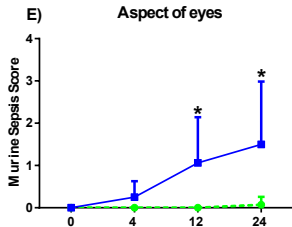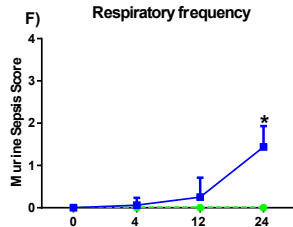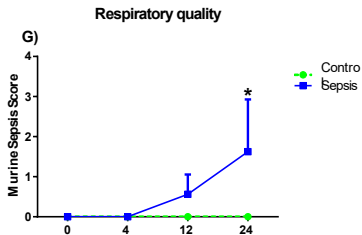

Supplement: Supplementary 2 — Figure S1: individualized MSS components, appearance, level of consciousness, activity, response to stimulus, aspect of eyes, respiratory frequency, and respiratory quality (A-G) within 24 hours after induction of sepsis with 20% fecal solution (1 mg/g). [file 5700853.f2.pdf]

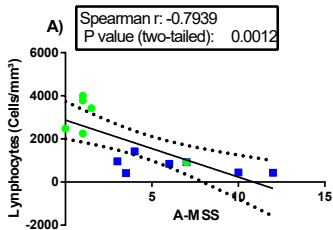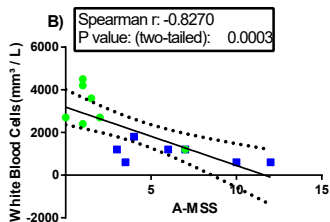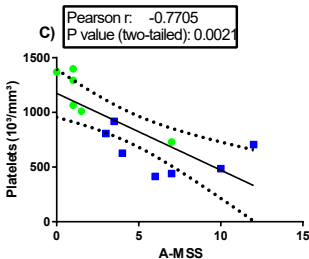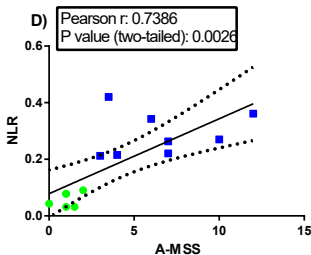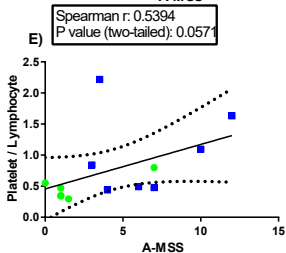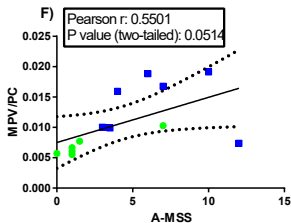

Supplement: Supplementary 3 — Figure S2: correlation between A-MSS four hours after induction and lymphocyte count, total leukocytes, platelets, and the ratios between neutrophils and lymphocytes (NLR), platelets and lymphocytes (PLR), and mean platelet volume and platelet count (MPV/PC) (A-F) within 24 hours after induction of sepsis with 20% fecal solution (1 mg/g). [file 5700853.f3.pdf]
